# Manipulating Levels of Socially Evaluative Threat and the Impact on Anticipatory Stress Reactivity

**DOI:** 10.3389/fpsyg.2021.622030

**Published:** 2021-02-22

**Authors:** Olivia A. Craw, Michael A. Smith, Mark A. Wetherell

**Affiliations:** ^1^Stress Research Group, Department of Psychology, Northumbria University, Newcastle upon Tyne, United Kingdom; ^2^Faculty of Medical Sciences, Population Health Sciences Institute, Newcastle University, Newcastle upon Tyne, United Kingdom

**Keywords:** stress, anticipation, social evaluation, multitasking, physiological stress response

## Abstract

Previous work suggests that relative increases in socially evaluative threat modulate the psychobiological stress response. However, few studies have compared stressors which manipulate the level of socially evaluative threat to which the participant is exposed. Here we present two studies. In the first, we assessed the integrity of an ecologically valid, laboratory stressor (direct socially evaluated multitasking) and its effects on acute psychobiological reactivity and ability to evoke an anticipatory response prior to participation. Specifically, we assessed whether the expectation and experience of direct social evaluation (multitasking while standing and facing an evaluator) evokes greater reactivity than indirect evaluation (over-the-shoulder evaluation). In the second study, we sought to replicate the findings regarding acute stress reactivity whilst extending the assessment window to assess the extent to which the stressor evokes anticipatory responses. As hypothesized, greater reactivity was observed following direct social evaluation compared with indirect observation. Increases in anxiety, heart rate and blood pressure were demonstrated across both studies and the paradigm therefore provides an ecologically valid technique for the activation of psychological and cardiovascular stress responding. Additionally, anticipation of experiencing socially evaluated multitasking led to increases in anxiety, tension, and worry prior to the event itself, supporting previous suggestions that threat anticipation may prolong the activation of stress mechanisms. In the present studies we assessed whether the expectation and experience of direct social evaluation evokes greater reactivity than indirect evaluation. The findings have demonstrated that direct social evaluation of multitasking is a more potent stressor than multitasking with indirect evaluation. Furthermore, our findings indicate that the period of anticipation of stressful events may be critical to understanding the process of stress regulation, and as such we recommend extending the sampling window to allow for the investigation of these processes.

## Introduction

Exposure to a situation perceived as challenging or threatening, which exceeds an individual's ability to cope, leads to a range of physiological responses which assist in managing the demand and mobilizing the required metabolic resources (Tomaka et al., 1993). When activated in the short-term these stress responses mediate adaption (McEwen, 2003); however, repeated or inappropriate activation is associated with a plethora of well-documented adverse effects on cardiovascular, immune, metabolic, and psychological health (McEwen, 1998). One of the determinants of vulnerability to stress-related ill-health is how individuals respond to daily stressors. Therefore, in order to understand the pathways by which exposure to stress leads to deleterious health outcomes it is necessary to develop tools which facilitate the observation of individuals while they are experiencing stress (Wetherell et al., 2006).

Previous studies have employed a variety of physiological and psychological stressors to assess responses of both the sympathetic adrenal medullary (SAM) and hypothalamic pituitary adrenal (HPA) axes, and provide an insight into their effects on health, cognition, and emotion (Schwabe et al., 2008). These stressors elicit a range of responses depending on their paradigm components; some activating the SAM axis, whilst others prompt activation of the slower reacting HPA axis. Whilst SAM reactivity is evidenced by more immediate responses to a stressor (such as cardiovascular reactivity and the release of adrenaline), HPA axis activity, detected through the secretion of cortisol from the adrenal glands, is typically observed in response to prolonged or more challenging (or resource-demanding) stressors. In particular, stressors that require motivated performance during social-evaluative conditions are associated with the most robust HPA reactivity in laboratory conditions (Kirschbaum et al., 1993; Dickerson and Kemeny, 2004). However, whilst many previously employed stressors have successfully elicited a range of stress responses, the development of a tool allowing desired procedural control, whilst also obtaining ecological validity, remains a challenge.

Beyond eliciting stress reactivity, laboratory stressors should provide insight into how an individual would respond to a stressor encountered in a real-life setting (Wetherell et al., 2006). Therefore, to achieve this ecological validity it is important that individuals engage with laboratory stressors representative of their experiences in natural settings. For example, daily activity typically involves exposure to multiple sources of stress (Chida and Hamer, 2008), and therefore single task stressors are of limited utility. Ecologically valid laboratory stressors should therefore encompass multiple stimuli in order to replicate the environments experienced in daily life (Wetherell et al., 2006). In addition to exposure to multiple sources of stress, many commonly encountered stressors include an interval of “preparing for” or “anticipating” the planned event (e.g., Neubauer et al., 2018). Studies examining the anticipatory response preceding forthcoming acute, naturalistic stressors suggest that this period is often perceived as highly stressful, and sometimes even more so than the event itself (e.g., Greco and Roger, 2003). Further, it has been suggested that this process can prolong the activation of stress mechanisms designed for short-term arousal only. For example, in studies assessing anxiety in patients attending hospital for surgery, orthopedic surgery patients reported the greatest levels of anxiety the day before hospital admission, 2 days prior to the operation (Johnston, 1980). A further study assessing patients awaiting surgery suggests that uncertainty and fear were more stressful for patients awaiting heart surgery than the symptoms of the heart condition itself (Bengtson et al., 1996). Similarly, women awaiting diagnoses following an abnormal mammogram described the waiting period as a type of “limbo” whereby their lives were seriously disrupted with “panic attacks, insomnia, inability to concentrate at work, inability to plan, gastrointestinal upset, tearfulness, and preoccupation of fears” (p. 45, Thorne et al., 1999). Finally, women undergoing an emergency ultrasound in early pregnancy reported significantly higher levels of anxiety before, compared with after the ultrasound (Richardson et al., 2017). This was even the case when they subsequently received a conclusive diagnosis, regardless of whether they received a positive or negative result, further demonstrating that anticipation may be perceived as a more potent stressor than the planned event itself.

The impact of forthcoming demand on resources has also been observed in non-patient settings. University students have reported greater anxiety during the period preceding final exams (Lotz and Sparfeldt, 2017) and greater levels of cortisol have been observed in the period preceding sport competitions (van Paridon et al., 2017; Cintineo and Arent, 2019) as well as in veterinary students prior to performing surgery (Stevens et al., 2019). Manipulations of forthcoming demand also provide evidence of the role of the HPA axis, specifically, the Cortisol Awakening Response (CAR) as an anticipatory mechanism. The CAR, a surge in cortisol in the period immediately following awakening is posited to play a role in preparation for forthcoming demand (Stalder et al., 2015). Increases in the magnitude of the CAR have been observed on the day of anticipated stress and demand in ambulatory (Wetherell et al., 2015) and sleep lab (Elder et al., 2018) settings.

Collectively, these findings indicate that real world stressors evoke “thinking about” the event in advance and anticipatory stress responses facilitate adaptive physiological functioning. The assessment of anticipatory periods could therefore be usefully incorporated into ecologically valid laboratory stressor paradigms to allow for the identification of biomarkers which may be associated with adaptive and maladaptive reactivity of stress response mechanisms.

In this paper we present two studies that demonstrate the development of such a paradigm. In the first study we investigate psychobiological reactivity related to an ecologically valid laboratory stressor, the Multitasking Framework (MTF: Purple Research Solutions, UK). The MTF is a motivated performance task that elicits stress through the manipulation of workload intensity and reliably activates indices of stress reactivity (Wetherell and Sidgreaves, 2005; Scholey et al., 2009; Kelly-Hughes et al., 2014; Allen et al., 2019). More recently we have demonstrated that multitasking, whilst being indirectly evaluated (over the shoulder feedback given on performance), leads to increased reports of anxiety and cardiovascular reactivity compared with multitasking alone (Wetherell et al., 2017). The effects of socially evaluative threat on psychobiological stress responses, particularly the impact upon HPA reactivity, are well-established (Dickerson et al., 2008, 2009). However, less is known about whether manipulations in the delivery of socially evaluative threat differentially impacts upon stress reactivity.

In Study 1 we aim to extend the findings of Wetherell et al. (2017), by manipulating the delivery of socially evaluative threat. We assess whether direct social evaluation of multitasking (i.e., participant standing and facing the researcher) leads to acute activation of the HPA axis, measured via the cortisol response to the stressor, relative to indirect social evaluation (i.e., researcher standing behind seated participant). We further aimed to investigate whether there was any evidence of anticipatory stress prior to stressor exposure. Study 2 presents a replication of the acute psychobiological reactivity to direct socially evaluated multitasking, whilst extending the sampling window to assess the day prior to, the day of, and the day following stressor exposure to capture psychobiological indices across an extended anticipatory period.

We hypothesized that (1) direct socially evaluated multitasking would elicit greater psychobiological stress reactivity than indirect socially evaluated multitasking, and (2) anticipation of experiencing direct socially evaluated multitasking would be associated with alterations in state mood and HPA reactivity prior to stress exposure.

## STUDY 1

### Materials and Methods

#### Participants

The sample comprised 39 healthy adults (age range 18–37, *M*_age_ = 22.0, *SD*_age_ = 4.62; 5 males, 34 females) from an undergraduate population. Participants were randomly allocated to receive either direct social evaluation (20 in total: three males, 17 females, *M*_age_ = 21.9, *SD*_age_ = 4.36) or indirect social evaluation (19 in total: two males, 17 females, *M*_age_ = 22.1, *SD*_age_ = 5.02) whilst multitasking. There were no significant differences between the two groups in terms of age, sex, perceived stress, or trait anxiety. Participants were screened for the following eligibility criteria: aged between 18 and 40 years, resting blood pressure <140/90 mmHg; not taking steroidal medication; not pregnant or breastfeeding; no history of panic attacks. All recruitment and study procedures were granted ethical approval from the Institutional Ethics Committee.

#### Materials

*The Multitasking Framework (MTF: Purple Research Solutions, UK)* is a computerized stressor that requires participants to attend to four tasks simultaneously that vary in terms of time pressure and/or difficulty; tasks are performance driven and demand is manipulated through instructing participants to achieve as high a score as they can. The present study included the following tasks: auditory monitoring, where participants are required to report a target tone; number tap, where participants are required to identify and report the highest digits in a 4 × 4 grid; visual monitoring, where participants are required to monitor and rest a cursor to prevent it leaving a target area; and a Stroop task (for a detailed description of tasks see Wetherell and Carter, 2014).

##### Cardiovascular Measurements

Heart rate and blood pressure were recorded using the DINAMAP Pro 200 V2 (GE Healthcare), via an inflatable brachial cuff.

##### Questionnaires

Items from the short-form State Anxiety scale (Marteau and Bekker, 1992*)* were used (I feel calm; I feel tense; I am upset; I feel relaxed; I feel content; I feel worried) alongside two additional items measuring stress (I feel stressed) and happiness (I feel happy) to assess state mood, with responses ranging from “not at all” to “very much.” Participants also completed items that assessed their feelings of mental alertness and physical tension in bed the night before, their levels of morning wellness and items to assess their thoughts about their forthcoming experiences (“to what extent have you been thinking about the stressor session?” and “to what extent have you been worrying about the stressor session?”).

##### Salivary Cortisol

Saliva samples were collected using Salivettes (Sarstedt, Germany). Samples were frozen (−20°C) and assayed in duplicate, using the enzyme-linked immunosorbent assay method (Salimetrics Europe, Cambridge UK, intra and inter assay coefficients <10%), in accordance with the manufacturer's instructions. Participants were requested to refrain from eating, drinking (other than water) and smoking for 1 h prior to providing saliva samples (Salimetrics Europe, Newmarket, United Kingdom).

#### Procedure

Participants satisfying the eligibility criteria were invited to provide written informed consent. Participants attended a 5-min study brief 2–7 days prior to their testing session. During this appointment direct social evaluation participants were notified that they would be required to stand in front of the researcher and receive social evaluation whilst completing a set of challenging tasks on the computer. Those in the indirect social evaluation condition were told that they would complete the tasks whilst seated and receive social evaluation from the experimenter behind them. Participants were provided with the state mood items and asked to complete them on the morning of the stressor session.

All laboratory testing took place at least 1 h following awakening and between 12:00 and 16:00 h, when levels of cortisol are typically lower and more stable (Saxbe, 2008). On the morning of the testing session (prior to attending the laboratory) participants completed the morning mood questionnaire. Upon arrival at the laboratory, participants were seated and the DINAMAP cuff was placed on their non-dominant arm. After a seated rest period of 10 min (Balodis et al., 2010), participants were given a 2-min demonstration of the tasks by the researcher (Wetherell and Carter, 2014). Prior to commencement of the 20-min period of multitasking, participants were informed that they needed to work as fast and accurately as possible, and to attain their highest achievable score. Upon cessation of the 20-min stressor participants were left alone in the room for a 20-min relaxation period. During this time, participants watched a nature documentary (Frozen Planet, BBC). In the indirect social evaluation condition, whilst completing the MTF participants received over-the-shoulder evaluation of their performance at set time intervals (see Wetherell et al., 2017, for full protocol). In the direct social evaluation condition, participants completed the MTF whilst standing behind a podium in front of the experimenter. The MTF screen was projected onto the wall behind them, enabling the researcher observation of participants' performance throughout the study. Social evaluation was received as per the indirect evaluation condition. Saliva was collected and state mood recorded at 5 intervals during the testing period: upon arrival at the laboratory; immediately before and after stressor exposure (20 min); 10 and 20 min following stressor cessation. Heart rate and blood pressure were recorded at the end of the rest period; following the MTF demonstration; and immediately, 10 and 20 min following stressor cessation. The procedure lasted 1 h. The protocol timeline and sampling procedure are presented in Table 1.

**Table 1:** Protocol timeline and sampling procedure: time (minutes) of each procedure prior/subsequent to the stress task displayed in the left column.

**Time (min)**	**Procedure**
−10	(Arrival at laboratory) Saliva sample 1 State mood 1 Heart rate (HR) and blood pressure (BP) measured, following rest period
−5	Demonstration of the MTF (2 min)
−3	Saliva sample 2 State mood 2 HR and BP
0	Stressor commencement HR and BP
+20	Stressor cessation Saliva sample 3 State mood 3 HR and BP
+40	Nature documentary commencement
+50	Saliva sample 4 State mood 4 HR and BP
+60	Nature programme cessation Saliva sample 5 HR and BP State mood 5

#### Treatment of Data

Mood, heart rate, blood pressure, and cortisol were assessed using 2-way repeated measures ANOVAs with group (direct social evaluation; indirect social evaluation) and time (Mood: arrival, post-stressor demonstration, stressor cessation, relaxation +10, relaxation +20/end of study; Cardiovascular parameters: arrival, post-stressor demonstration, stressor cessation, relaxation +10, relaxation +20/end of study; Cortisol: arrival, pre-stressor, post-stressor, relaxation +10, and relaxation +20/end of study). Bonferroni-corrected *post-hoc* analyses were conducted to assess changes between the 5 time points (arrival; post-stressor demonstration; stressor cessation; relaxation +10; relaxation +20/end of study).

### Results

#### Cardiovascular Parameters

**Figure 1:**
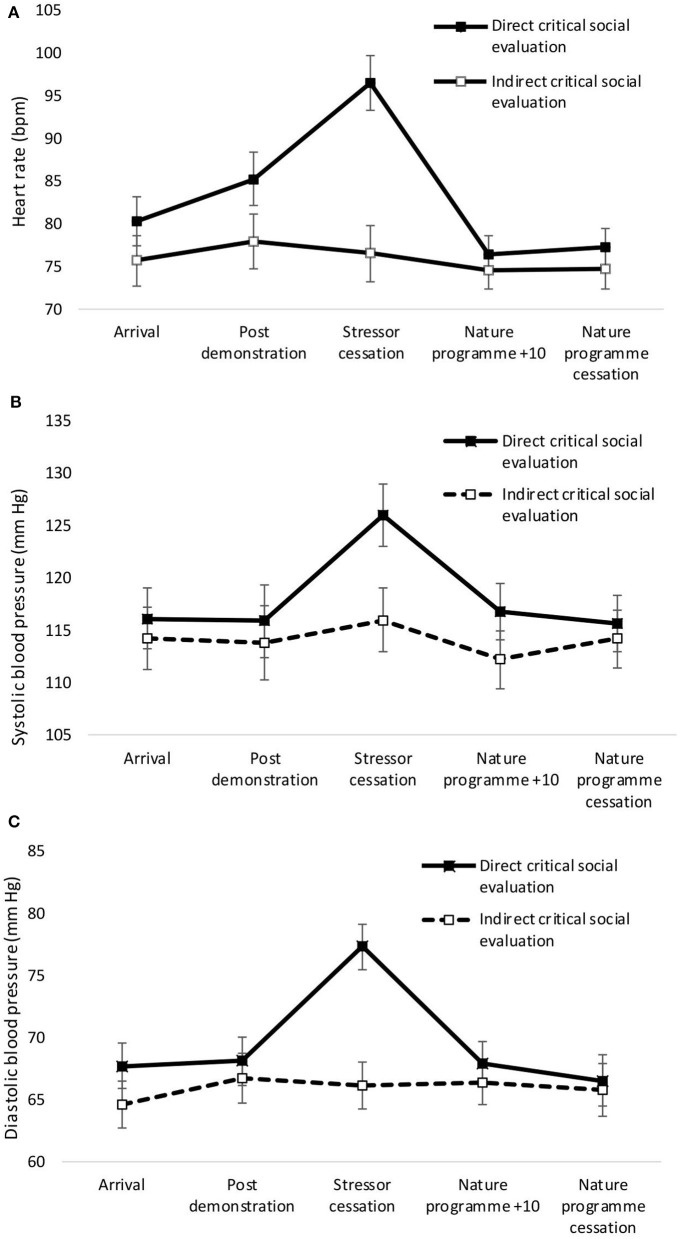
Mean (and SE) heart rate **(A)**, systolic **(B)**, and diastolic **(C)** blood pressure for direct and indirect social evaluation conditions.

A significant main effect of condition was observed for heart rate, with greater heart rate for the direct compared to the indirect condition [*F*_(1,37)_ = 4.29, *p* = 0.045, η^2^ = 0.10]. There was no significant main effect of condition for systolic [*F*_(1,37)_ = 1.166, *p* = 0.287, η^2^ = 0.03] or diastolic [*F*_(1,37)_ = 2.63, *p* = 0.113, η^2^ = 0.07] blood pressure. Significant main effects of time were observed for heart rate [*F*_(4,34)_ = 13.56, *p* < 0.001, Wilks' Λ = 0.39, η^2^ = 0.66, see Figure 1A]. Pairwise comparisons revealed significant increases in heart rate from arrival at the laboratory to stressor cessation (*p* < 0.001). There was a significant main effect of time on systolic blood pressure [*F*_(4,34)_ = 3.27, *p* = 0.023, Wilks' Λ = 0.72, η^2^ = 0.28, see Figure 1B]. Pairwise comparisons revealed a significant increase in systolic blood pressure from arrival to stressor cessation (*p* = 0.032). A significant main effect of time on diastolic blood pressure was observed [*F*_(4,34)_ = 5.45, *p* = 0.002, Wilks' Λ = 0.61, η^2^ = 0.39, see Figure 1C]. Pairwise comparisons revealed a significant increase from arrival at the laboratory to stressor cessation (*p* = 0.001). A significant condition × time interaction was observed for heart rate [*F*_(4,34)_ = 8.95, *p* = <0.001, Wilks' Λ = 0.49, η^2^ = 0.17] and diastolic blood pressure [*F*_(4,34)_ = 4.66, *p* = 0.004, Wilks' Λ = 0.65, η^2^ = 0.35]. A one-way ANOVA confirmed that cessation of the stressor was the point at which heart rate [*F*_(1,37)_ = 26.29, *p* = <0.001, η^2^ = 0.42], systolic blood pressure [*F*_(1,37)_ = 4.89, *p* = 0.033, η^2^ = 0.12], and diastolic blood pressure [*F*_(1,37)_ = 19.67, *p* < 0.001, η^2^ = 0.35] significantly differed between the groups.

Mean (and S.E.) heart rate, systolic and diastolic blood pressure across both conditions are presented in Figure 1.

#### State Mood

**Figure 2:**
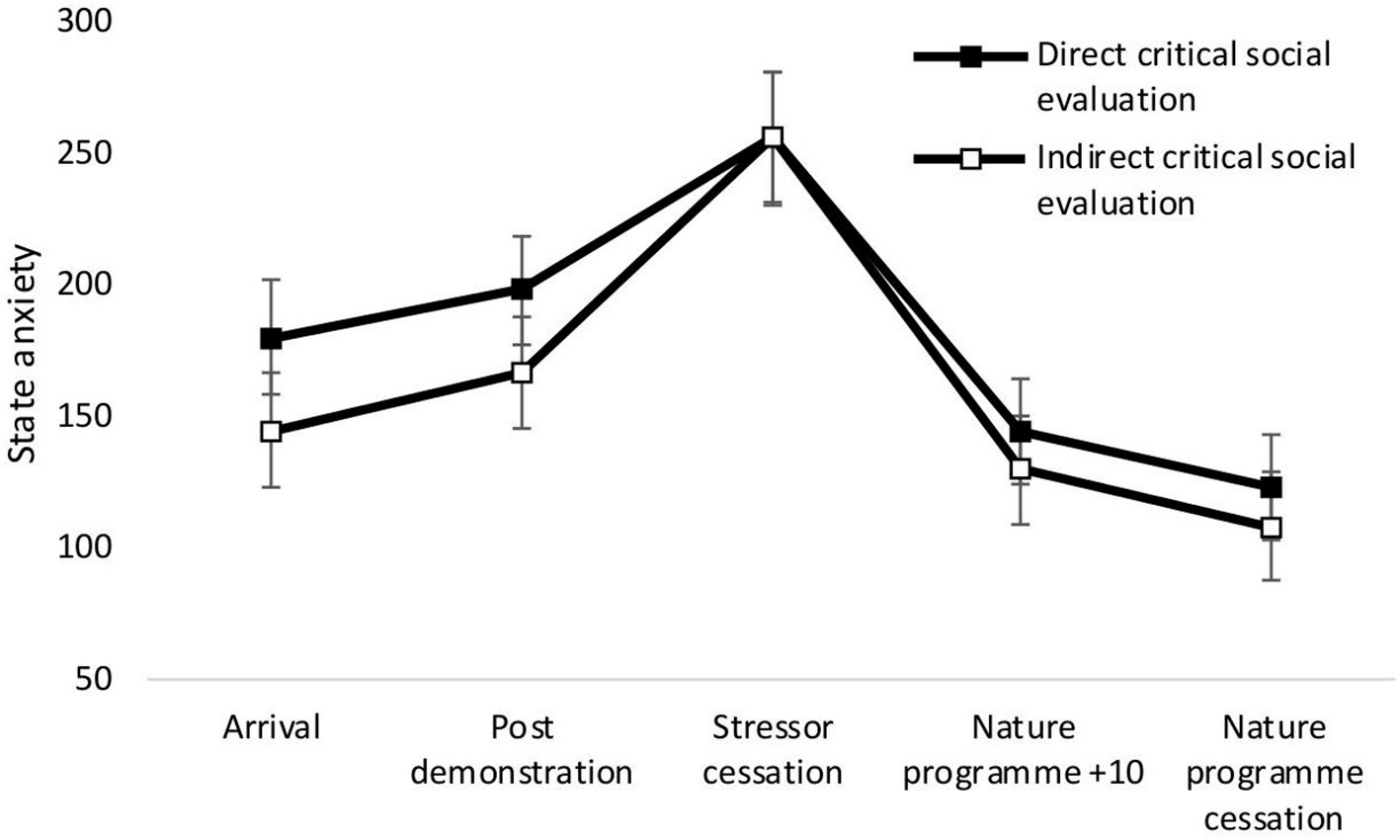
Mean (and SE) state anxiety for the direct social evaluation and indirect social evaluation conditions, assessed during exposure to the stressor manipulation.

There was a significant main effect of time on state anxiety [*F*_(4,34)_ = 27.36, *p* < 0.001, Wilks' Λ = 0.24, η^2^ = 0.76, see Figure 2]. Pairwise comparisons revealed a significant increase in anxiety from arrival at the laboratory and stressor cessation (*p* < 0.001) and decrease between arrival and nature documentary cessation (*p* = 0.001). No significant main effects of condition [*F*_(1,37)_ = 0.50, *p* = 0.485, η^2^ = 0.01], or time × condition interactions were observed [*F*_(4,34)_ = 0.50, *p* = 0.739, Wilks' Λ = 0.95, η^2^ = 0.06]. See Figure 2.

#### Cortisol

A significant main effect of time was observed for cortisol, representing a reduction across the testing period [*F*_(4,29)_ = 6.35, *p* = 0.001, Wilks' Λ = 0.53, η^2^ = 0.47]. There was no significant main effect of condition [*F*_(1,32)_ = 0.41, *p* = 0.527, η^2^ = 0.01] or time × condition interaction [*F*_(4,29)_ = 1.81, *p* = 0.154, Wilks' Λ = 0.80, η^2^ = 0.02].

In order to adjust for the potential confounding influence of diurnal cortisol on cortisol reactivity, salivary cortisol values at each time point were adjusted for the diurnal slope in accordance with the procedure outlined by Elbau et al. (2018) and Kühnel et al. (2020), and the area under the curve was calculated using these adjusted values. There was no significant difference between the direct and indirect social evaluation conditions using this measure of cortisol reactivity [*t*_(32)_ = 0.17, *p* = 0.86, *d* = 0.06].

#### Awakening Mood on Day of Stressor

Participants in the direct social evaluation condition reported significantly greater feelings of tension on the morning of the stressor, compared with those who were expecting indirect social evaluation [*F*_(1,37)_ = 6.12, *p* = 0.018, η^2^ = 0.14]. They additionally reported feeling less content [*F*_(1,37)_ = 5.79, *p* = 0.021, η^2^ = 0.17], less calm [*F*_(1,37)_ = 8.40, *p* = 0.006, η^2^ = 0.18], and less happy [*F*_(1,37)_ = 6.54, *p* = 0.015, η^2^ = 0.15] than those exposed to indirect social evaluation.

## STUDY 2

### Method

#### Participants

The sample comprised 31 healthy adults (age range 18–38 years, *M*_*age*_ = 24.4, *SD*_*age*_ = 5.18) from an undergraduate population (none of whom had taken part in Study 1). One participant withdrew from the study after the first sampling day due to sleep disturbance. Thirty participants remained in the final analysis (10 males, 20 females).

#### Materials

All materials were identical to those used in Study 1.

#### Procedure

Participants satisfying the eligibility criteria (reported in Study 1) were invited to the laboratory for a short (10 min) briefing 2–4 days prior to the stressor session. Participants were told that they would be completing a stressor task which would be cognitively demanding, whilst standing in front of a researcher who would be monitoring their behavior and performance throughout the task and that they would receive critical evaluation of their performance throughout. Following receipt of written informed consent, participants were given data collection packs to take away with them. These packs included four sets of salivettes, a questionnaire booklet including state mood measures and a saliva sample collection diary. Participants received training on how to provide the saliva samples and were instructed to accurately record saliva collection times. Additionally, participants were asked to wear wrist-worn actigraph devices for the duration of the study and were told that their waking times were recorded. Unfortunately, problems with data recording prevented analyses of these data. Participants were asked to provide saliva samples immediately upon awakening; 30, 45, and 60 min following awakening; 6 h following awakening and immediately prior to bed on 3 consecutive days: the day before returning to the laboratory for the stressor (day 1), the day of the stressor (day 2), the day after (day 3), and on a control day (day 4). Additionally, participants recorded state mood upon awakening, 6 h after awakening and before bed and recorded the times at which they woke and provided each saliva sample. The Study 1 protocol for direct social evaluation was replicated. Participants provided all completed saliva samples upon arrival at the laboratory and returned the final day of samples after completion.

#### Treatment of Data

For acute stress reactivity, mood, heart rate, blood pressure, and cortisol were assessed using one-way repeated measures ANOVAs (variables and time points as in Study 1). Participants with missing cortisol data were excluded from acute cortisol analysis.

Daily variation in mood was assessed using two-way repeated measures ANOVAs with day (pre-stressor, stressor, post-stressor, control) and time (waking, waking +6 h, bed time). CAR magnitude was assessed using a repeated measures ANOVA (day: pre-stressor, stressor, post-stressor, control), and was expressed as the increase in cortisol observed between waking and the maximum secretion post-awakening.

Diurnal cortisol secretion was assessed by area under the curve with respect to ground (AUCg). AUCg was calculated for each participant on each day using the cortisol level (nmol/l) at each sampling point and the time (min) between each sample (Pruessner et al., 2003). Diurnal cortisol secretion was not calculated for participants who did not provide sufficient samples for AUCg calculation. Therefore, due to missing samples (insufficient saliva volume and/or missing samples) considerably reducing sample size for this analysis, AUCg analysis was conducted for the pre-stressor and stressor days only.

### Results

#### Stressor Effects

##### Cardiovascular Parameters

**Figure 3:**
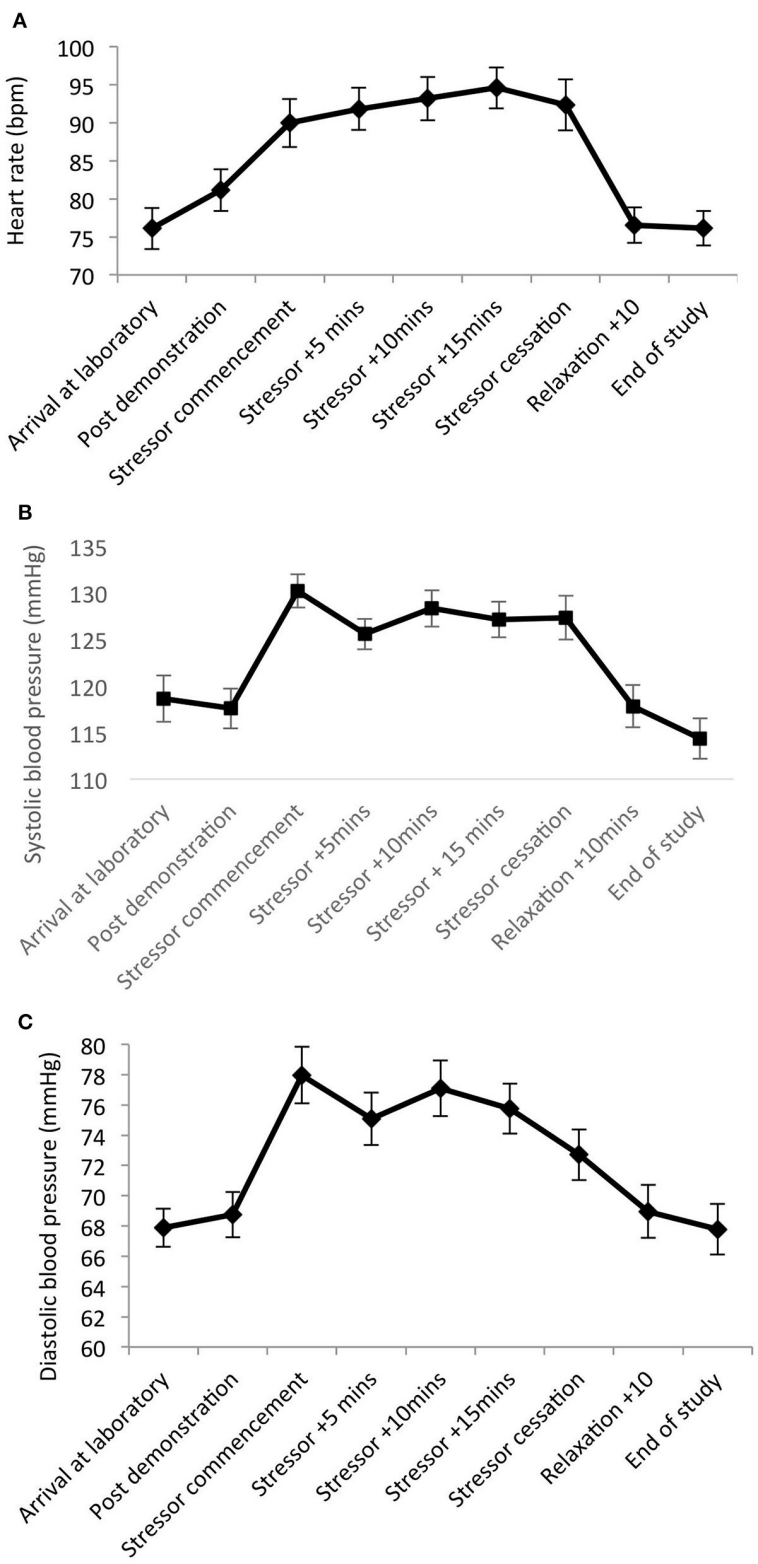
Mean (and SE) heart rate **(A)**, systolic **(B)**, and diastolic **(C)** blood pressure assessed during exposure to the stressor manipulation.

Significant main effects of time were observed for heart rate [*F*_(8,20)_ = 19.82, *p* < 0.001, Wilks' Λ = 0.11, partial η^2^ = 0.89]; SBP [*F*_(8,20)_ = 15.31, *p* < 0.001, Wilks' Λ = 0.14, η^2^ = 0.86]; and DBP [*F*_(8,20)_ = 5.36, *p* = 0.001, Wilks' Λ = 0.32, η^2^ = 0.68]. Pairwise comparisons revealed significant increases from arrival at the laboratory, to during and post-stress measures across all parameters (*p* = 0.001–0.03). See Figure 3.

##### State Anxiety

**Figure 4:**
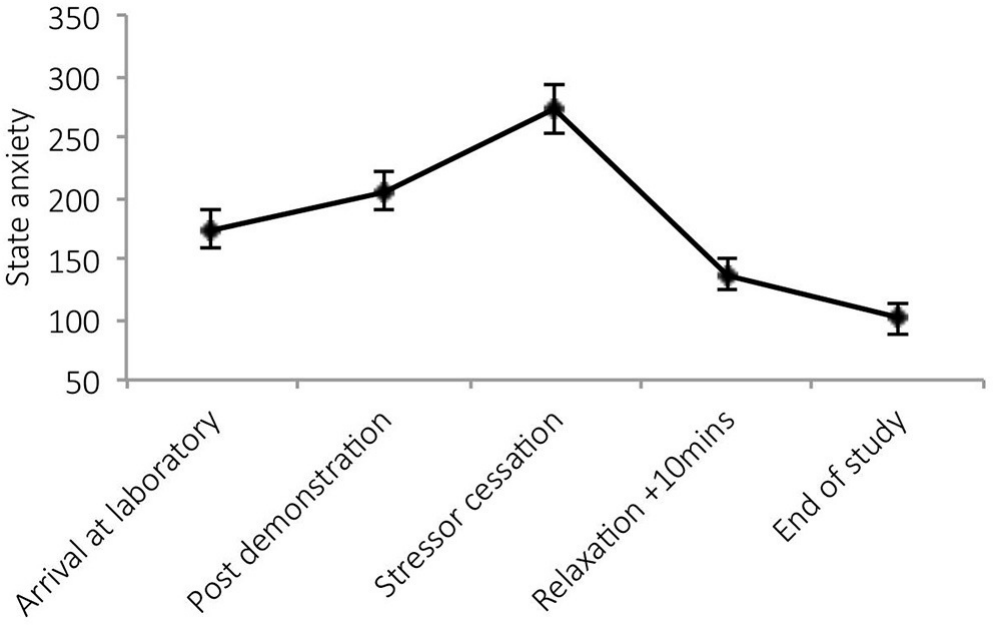
Mean (and SE) state anxiety, assessed during exposure to the stressor manipulation.

There was a significant main effect of time on state anxiety [*F*_(4,23)_ = 19.03, *p* < 0.001, Wilks' Λ = 0.23, η^2^ = 0.77]. *Post-hocs* revealed significant increases in anxiety post-stressor demonstration (*p* = 0.02) and post-stressor (*p* < 0.001) and reductions during relaxation (*p* = 0.01). See Figure 4.

##### Cortisol

There were no significant changes in cortisol across the stressor period [*F*_(4,21)_ = 1.96, *p* = 0.138, Wilks' Λ = 0.73, η^2^ = 0.27].

#### Non-acute Variables

##### Psychological Indices

**Figure 5:**
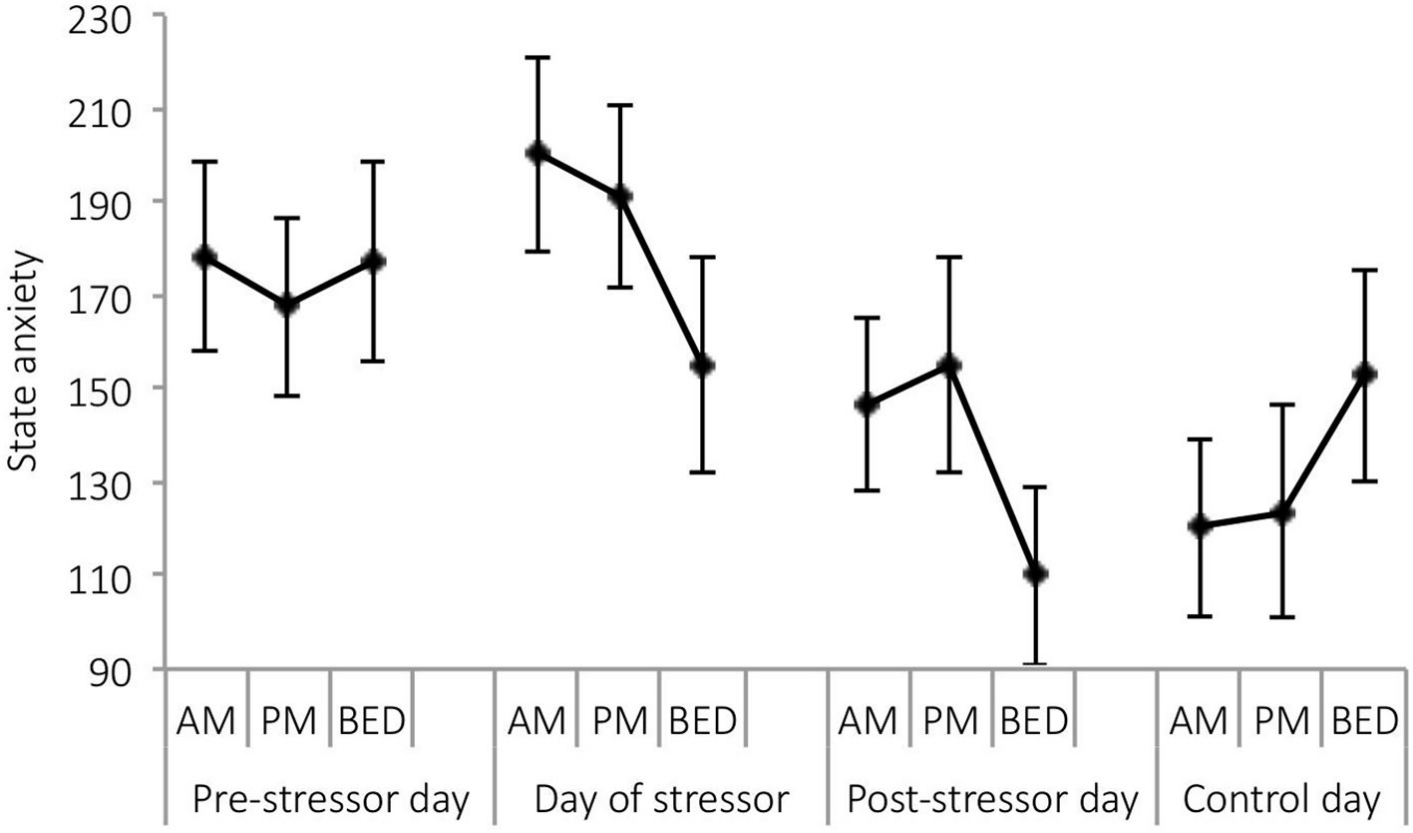
Mean (and SE) state anxiety assessed in the morning, at waking +6 h and bedtime, over the four sampling days.

A significant main effect of day was observed for state anxiety [*F*_(3,21)_ = 4.05, *p* = 0.020, Wilks' Λ = 0.63, η^2^ = 0.37]. Pairwise comparisons revealed significantly greater reports of state anxiety on the day of the stressor, compared with the post-stressor day (*p* = 0.024), and control day (*p* = 0.026). See Figure 5.

**Figure 6:**
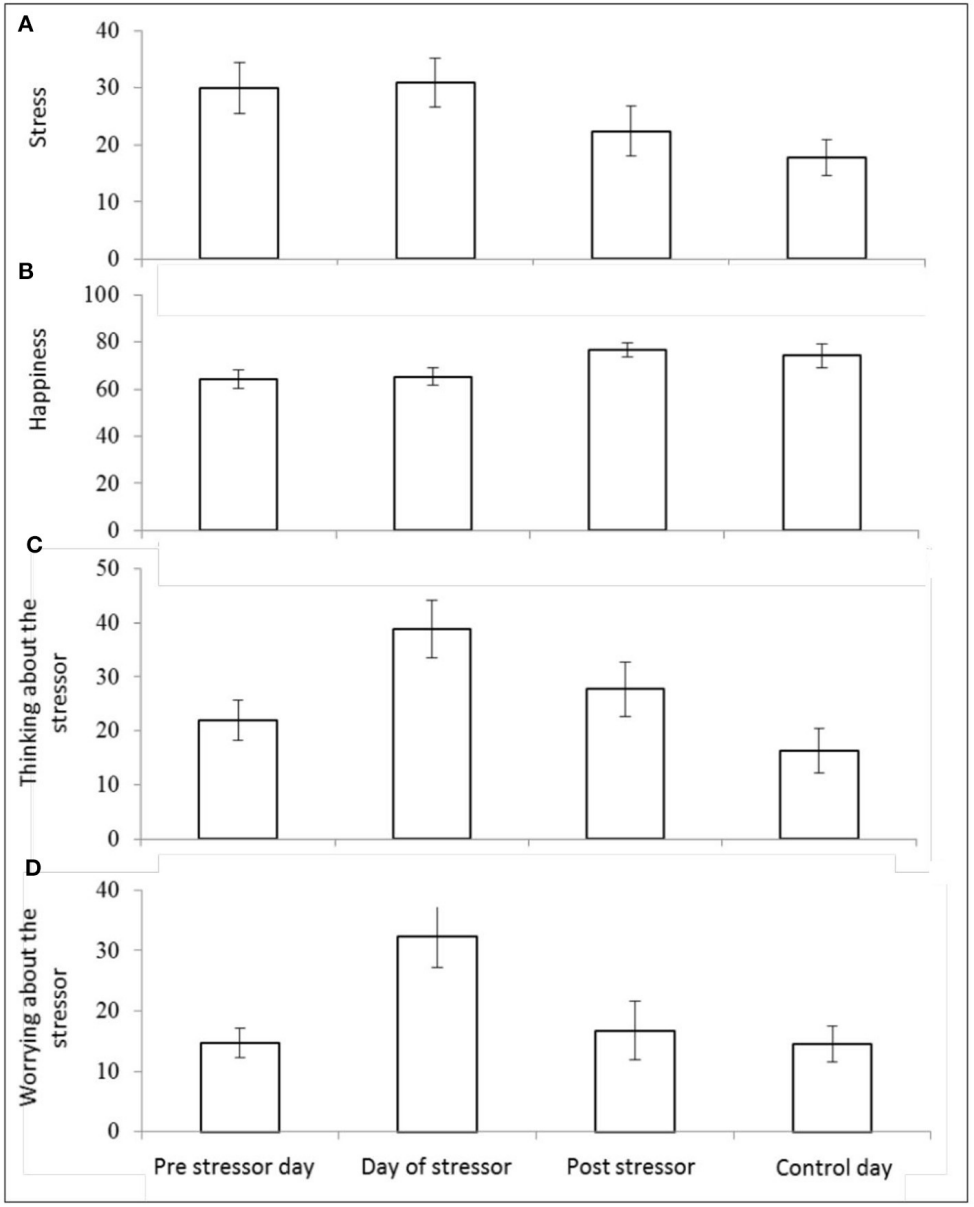
Mean (and SE) morning self-reported stress **(A)**, happiness **(B)**, “thinking about” the stressor **(C)**, and “worrying about” the stressor **(D)**, assessed over four sampling days.

There was a significant main effect of day on self-reported stress [*F*_(3,25)_ = 4.32, *p* = 0.014, Wilks' Λ = 0.66, η^2^ = 0.34]. Pairwise comparisons revealed significantly greater reports of stress on the pre-stressor day compared with control day (*p* = 0.009), day of stressor compared with post-stressor day (*p* = 0.040), and the day of the stressor compared with the control day (*p* = 0.004). See Figure 6A.

There was also a significant main effect of day on self-reported happiness [*F*_(3,25)_ = 4.34, *p* = 0.014, Wilks' Λ = 0.66, η^2^ = 0.34]. Pairwise comparisons revealed significantly higher scores for happiness reported on: post-stressor day compared with pre-stressor day (*p* = 0.006), the control day compared with the pre-stressor day (*p* = 0.040), and on the post-stressor day compared with the day of the stressor (*p* = 0.005). See Figure 6B.

There was also a significant main effect of day on the extent to which participants reported (in the morning) thinking about the stressor session [*F*_(3,24)_ = 6.33, *p* = 0.003, Wilks' Λ = 0.56, η^2^ = 0.44]. Pairwise comparisons revealed significantly greater reports of thinking about the stressor session on: the day of the stressor compared with the pre-stressor day (*p* = 0.004), the day of the stressor compared with the post-stressor day (*p* = 0.048), the post-stressor day compared with the control day (*p* = 0.010), and the day of the stressor compared with the control day (*p* = <0.001). See Figure 6C.

There was a significant effect of day on self-reported worrying about the stressor [*F*_(3,20)_ = 4.50, *p* = 0.014, Wilks' Λ = 0.60, η^2^ = 0.40]. Pairwise comparisons revealed significantly greater reports of worrying on the morning of the stressor compared with the pre-stressor day (*p* = 0.001) the day of the stressor compared with the post-stressor day (*p* = 0.006), and the day of the stressor compared with the control day (*p* = 0.003). See Figure 6D.

#### Basal Cortisol Indices

##### Diurnal Secretion

A paired samples *t*-test revealed no significant differences in AUC_G_ between pre-stressor day (*M* = 5816.67, *SD* = 3753.40) and day of stressor (*M* = 5751.38, *SD* = 2959.98), *t*_(16)_ = 0.12, *p* = 0.909.

##### CAR Magnitude

There were no significant differences in CAR magnitude [*F*_(3,18)_ = 1.50, *p* = 0.248, Wilks' Λ = 0.80, partial η^2^ = 0.20], across the days: pre-stressor day (*M* = 8.57, *SD* = 8.58); day of stressor (*M* = 6.44, *SD* = 7.95); post-stressor day (*M* = 3.55, *SD* = 6.78); control day (*M* = 5,99, *SD* = 6.89).

## Discussion

The aims of this paper were 2-fold: firstly, to present the development of an ecologically valid laboratory stressor (direct socially evaluated multitasking) and its effects on acute psychobiological reactivity and its ability to elicit anticipatory responses prior to participation. Specifically, we assessed whether the expectation and experience of direct social evaluation evokes greater reactivity than the indirect social evaluation reported previously (Wetherell et al., 2017). Secondly, we sought to replicate the findings regarding acute stress reactivity whilst extending the assessment window to assess the extent to which the stressor evokes anticipatory responses. In relation to the first aim, as hypothesized, greater reactivity was observed following direct social evaluation compared with indirect observation. Specifically, direct social evaluation elicited significantly greater heart rate and diastolic blood pressure reactivity. This pattern also emerged for systolic blood pressure; however, the difference between the stressors was not statistically significant. This may reflect differences in the underlying mechanisms involved in physiological functioning (e.g., Allen et al., 1987; Willemsen et al., 1998) or the modest samples size being underpowered to detect between group differences. The large effect sizes and the demonstration of significant reactivity of all cardiovascular measures in Study 2 suggest the latter.

In addition to increased cardiovascular activity as a function of direct social evaluation, other differences between the conditions should also be considered. Specifically, changes in orthostatic pressure from seating to standing may also contribute to the greater cardiovascular reactivity observed in the direct socially evaluated condition. That is, unlike the indirect social evaluation condition where participants remain seated throughout, the direct social evaluation condition required participants to stand for the duration of the stressor and this process can lead to increases in cardiovascular parameters (e.g., Freeman et al., 2011). It is therefore important to acknowledge that these orthostatic changes may have additional effects on cardiovascular responses over and above that of direct social evaluation. This issue however is not specific to the current paradigm, indeed the current cardiovascular measurement protocol was based on the standard Trier Social Stress Test (TSST) procedure where following a period of seated baseline, participants complete the stressor task while standing (e.g., Kirschbaum et al., 1993).

The cardiovascular reactivity observed across both studies is consistent with previous studies using similar measurement protocols comprising social evaluation (e.g., TSST: Allen et al., 2014). Taken together, these provide further evidence that social observation during performance tasks elicits physiological reactivity; however, social evaluation elicited by direct observation is a more potent stressor than indirect, over-the shoulder evaluation. This supports the notion that situations involving a perceived threat to the “social self,” as would be experienced through direct evaluation of multitasking performance, are potent stressors (Dickerson and Kemeny, 2004; Dickerson et al., 2009).

In line with this notion, we hypothesized that direct social evaluation would also elicit acute cortisol reactivity. This, however, was not the case even following statistical adjustment for the impact of the diurnal decline across this assessment period. The stressor task was developed to incorporate the key components present in laboratory stressors: motivated performance, uncontrollability, and social evaluation. Moreover, the paradigm was manipulated to increase the level of social evaluation, experienced through delivering social feedback directly to the participant, rather than over the shoulder. The absence of cortisol reactivity may therefore be explained by evaluating how this stressor paradigm differs from other stressors that reliably elicit cortisol reactivity, specifically, the TSST (Kirschbaum et al., 1993). The TSST requires participants to deliver a free speech and verbal mental arithmetic for 10 min to a panel of assessors following a 5 min preparation period. In common with the TSST, the current stressor involves the completion of challenging tasks; however, the responses are entered via a computer rather than vocalized to a panel of assessors. As other stressors have demonstrated HPA reactivity to single and even virtual assessors (Turner-Cobb et al., 2019), it seems likely that public speaking plays a crucial role in activating the HPA axis. Public speaking presents a potent threat to the “social self” and carries a significant risk of social embarrassment and humiliation leading to distress (Garcia-Leal et al., 2014). As such, public speaking is a well-documented stressor, causing cardiovascular activation (Elsenbruch et al., 2006), and cortisol reactivity (Auer et al., 2018). HPA reactivity has been observed in some individuals in response to other stressors that do not comprise a verbal component (e.g., the imaging stress task used within an MRI scanner, Kühnel et al., 2020). However, the absence of a public speaking element may be an explanation for the lack of salivary cortisol reactivity in response to the current stressor.

Both stressors increased state anxiety, and this finding was replicated for direct observation in Study 2. However, there were no differences in acute anxiety responses between direct and indirect social evaluation. This finding is also consistent with Dickerson and Kemeny (2004) who report that socially evaluative stress may elicit different patterns of physiological reactivity but may not increase self-reports of distress. Moreover, they suggest that socially evaluative stress may lead to more specific cognitive and emotional reactions. In support, we observed changes in a number of emotional responses in anticipation of completing a socially evaluative stressor. Those who anticipated forthcoming direct social evaluation reported feeling more tense, and less calm, content and happy on the morning of the stressor than those expecting to experience indirect social evaluation. These differences suggest that the appraisal mechanics engaged during stressor exposure may also be engaged during the anticipatory period prior to exposure (Everly and Lating, 2019). This concept was therefore assessed more comprehensively in Study 2. In support, participants reported significantly greater state anxiety and stress on the day of the stressor than the day following. Participants also reported both “thinking about” and feeling significantly more worried about the stressor session on the morning of the laboratory session compared to both the day prior and post-stressor exposure. This finding is consistent with previous work investigating anticipation of a socially evaluative laboratory stressor, whereby participants reported greater levels of tension, perceived stress, and anxiety on the morning of the anticipated stressor (Wetherell et al., 2015). Unlike this study, however, anticipation of the stressor in the current study did not alter cortisol secretion following awakening (CAR) or levels across the day (AUCg). Although in both studies participants were expecting a socially evaluative threat, public speaking may have been perceived as a greater threat than the observed multitasking anticipated in the current study. It has been argued that the cognitive preoccupation with upcoming tasks acts as a stressor in its own right (Ennis et al., 2001; Schlotz et al., 2004) and can, therefore, subsequently promote HPA axis activation (Roger and Najarian, 1998).

We have previously discussed the adaptive role of the anticipatory stress response in preparing for forthcoming demand, with specific attention to observing greater anticipatory reactivity prior to planned threat and challenge. However, the absence of anticipatory cortisol responses in this study may also be considered from an evolutionary perspective; the ability to conceptualize and accurately anticipate potential challenges or threats is adaptive, as the negative affect associated with anticipating a stressful event allows for appropriate modifications to be made with regards to behavior, cognition and physiology. This process means the individual is prepared for the forthcoming demand, but in some situations the forward-planning this requires, combined with the motivation to take measures to avoid the event, could result in mitigating the threat altogether (Aspinwall and Taylor, 1997; Schulkin, 2011), or merely appraising the event to be less threatening, due to having time to prepare. Dickerson and Kemeny (2004) describe a dose-response relationship between the degree of social evaluation involved in a stressor, and the magnitude of cortisol response, and therefore the degree to which the event is deemed “threatening” has a direct impact on the subsequent response. The earlier examples reporting greater cortisol in the period preceding events (e.g., sport competitions; van Paridon et al., 2017; Cintineo and Arent, 2019; veterinary students performing surgery; Stevens et al., 2019) were more personal with greater perceived consequences. In comparison, the threat of forthcoming social evaluated multitasking may have been sufficient to modify emotional responses, but not potent enough to alter HPA function.

These findings should be considered in light of limitations. First, the sample sizes were of sufficient size to observe predicted changes in the psychobiological variables that have been previously evaluated in relation to this paradigm. However, other related issues should be considered, in particular, the majority of participants were female. There are well-documented sex differences in stress responding, males typically demonstrate greater cortisol reactivity than females (Kudielka et al., 2009); females produce greater cortisol responses during the luteal than during the follicular phase of the menstrual cycle (Childs et al., 2010); and hormonal contraceptive use may also contribute to variability in stress reactivity (Nielsen et al., 2013). Our sample size, compounded by instances of missing menstrual cycle data prevented meaningful analyses to correct for these factors, and potential differences related to the sample should therefore be acknowledged. Second, other individual differences may have impacted upon stress reactivity. For example, although the Multitasking Framework is performance driven, actual performance was not recorded. It may be the case that better performance would impact upon stress reactivity; however, the robust increases in psychological and cardiovascular stress reactivity demonstrate the capacity of the paradigm to induce stress. Finally, the assessment of the CAR relies upon accurate sampling and adherence to the sampling protocol, particularly the timing of waking in relation to the provision of samples. In line with guidelines (Stalder et al., 2015), strategies were implemented to facilitate protocol adherence; participants were given full training on appropriate collection, were made aware of the importance of following the protocol and were asked to provide a record of waking and sample provision times. Attempts were made to objectively monitor waking and sampling times through wrist-worn actigraphy devices, unfortunately, these data could not be used as intended. The wearing of the devices alongside information regarding monitoring can increase adherence (Broderick et al., 2004), and in support, there were no instances of non-adherence based on participants' self-reported timings. However, the absence of objective monitoring to verify participant waking and sample provision may impact upon the accuracy samples obtained during the CAR period.

Notwithstanding these limitations, the present studies have demonstrated that direct social evaluation of multitasking is a more potent stressor than multitasking with indirect social evaluation. The stressor does not involve public speaking, and this is a likely explanation for the absence of cortisol reactivity. Nonetheless, increases in anxiety, heart rate and blood pressure were demonstrated across two studies and the paradigm therefore provides an ecologically valid technique for the activation of psychological and cardiovascular stress responding. Additionally, anticipation of experiencing socially evaluated multitasking led to alterations in state factors prior to the event itself. Although the personal salience of the forthcoming task is a likely explanation of the absence of HPA reactivity during this period, changes in state mood and cognitive appraisal provide evidence that the period prior to forthcoming events is subject to anticipatory processes. The period of anticipation of stressful events may be critical to understanding the process of stress regulation (Ottaviani, 2018), and as such, we recommend the use of paradigms that extend the sampling window around an anticipated stressor to allow for the investigation of these processes.

## Data Availability Statement

The original contributions presented in the study are included in the article/Supplementary Material, further inquiries can be directed to the corresponding author/s.

## Ethics Statement

The studies involving human participants were reviewed and approved by Department of Psychology, Faculty of Health and Life Sciences, Northumbria University Newcastle. The patients/participants provided their written informed consent to participate in this study.

## Author Contributions

OC, MS and MW designed the study protocols and prepared the manuscript. OC executed the study protocols and ran the preliminary analyses. All authors contributed to the article and approved the submitted version.

## Conflict of Interest

The authors declare that the research was conducted in the absence of any commercial or financial relationships that could be construed as a potential conflict of interest.
